# RISK-TAKING BEHAVIOUR AND EXECUTIVE FUNCTIONS, A MAJOR COMPONENT OF THE RISK OF FALL FACTORS AFTER RECENT STROKE

**DOI:** 10.2340/jrm.v56.40153

**Published:** 2024-10-22

**Authors:** Alain P. YELNIK, Ines DEKIMÈCHE, Emna JELILI, Ioannis BARGIOTAS, Marylène JOUSSE, Johann BEAUDREUIL, Alexis SCHNITZLER

**Affiliations:** 1 Physical and Rehabilitation Medicine Department, GHU APHP-Nord, Université Paris Cité, Paris; 2 Centre Borelli, Gif-sur-Yvette, France

**Keywords:** behaviour, executive functions, fall, stroke

## Abstract

**Objective:**

This study investigated the weight of different cognitive disorders on patient behaviour influencing the risk of falls after recent stroke.

**Design:**

Survey and retrospective monocentric study.

**Subjects/patients:**

74 professionals/108 patients.

**Methods:**

Survey of professionals to ask for their thoughts concerning the weight of different cognitive disorders on the risk of falls and a retrospective study of patients post-stroke to determine whether these cognitive deficits could distinguish fallers from non-fallers. Univariate and multivariate logistic regression analyses were conducted.

**Results:**

In part 1, major cognitive disorders identified were anosognosia, confusion, inattention, precipitation, and unilateral spatial neglect. In part 2, 25 patients (23%) were fallers. After adjustment for length of rehabilitation stay and disease severity, on multivariate analysis, the cognitive disorders significantly associated with risk of falls were anosognosia (odds ratio 16), precipitation (13.3), inattention (8.3), and perseveration (4.9). Unilateral spatial neglect was not independently associated. Aphasia did not play a role.

**Conclusion:**

Some cognitive disorders, easily identified before any neuropsychological assessment, strongly modify patient behaviour in terms of risk of falls. It is proposed that these disorders should not be considered as an additional factor along with physical and general factors but rather as a multiplying factor applied to the others.

## LAY ABSTRACT

After stroke, the frequency of falls is high. Being able to anticipate the risk for an individual is a constant challenge for rehabilitation teams because stimulation of recovery needs to encourage patients to walk despite balance disorders. The behaviour of patients is one of the main risk factors for fall. The risk depends on certain cognitive capacities, often underestimated, which should be recognized as soon as possible, before any neuropsychological assessment. This study involved a survey of 74 professionals to identify the main cognitive disorders perceived as responsible for behaviour that might lead to a fall, then a retrospective study of 108 patients investigating whether they could distinguish fallers from non-fallers. Anosognosia, precipitation, inattention, and perseveration were strongly associated with the risk of fall. These cognitive disorders should not be considered as an additional factor along with physical and general factors but rather as a multiplying factor applied to the others.

After stroke, the frequency of falls is high. During the first hospital stay, the number of reported fallers in an acute stroke unit ranges from 8.5% (1) to 13% (2) and in a rehabilitation centre from 16% (3), 21.8% (4), and 36% (5) to 37% (6). During the 6 months after stroke or discharge, the proportion ranges from 45% (7) and 58% (8) to 73% (9) and during the first year after stroke from 42.8% (10) and 55% (11) to 57.6% (12).

Being able to anticipate the risk of falls for an individual is a constant challenge. Avoiding any risk by forbidding walking alone is efficient, but the only way to improve balance is by training balance during physical therapy exercises and usual daily activities. Hence, in a physical medicine and rehabilitation (PRM) department, the individual must be encouraged to be active alone. This attitude is a risk-taking behaviour, involving the whole team. It supposes knowing the individual’s risk of falling, which cannot be predicted by any test or score. The risk of falls should be estimated on the basis of a network of arguments: all the disorders of balance control including bias of verticality, paralysis, or lack of sensory information as well as general or contextual factors, including cognitive disorders.

However, the impact of cognitive disorders on the risk of falls is often not studied. Only 15 of the 27 studies of a recent review investigated this issue, with 11 reporting a relation (13). The results could have depended on how the cognitive disorders were defined. With general measures such as the Mini-Mental State Examination (MMSE) (8, 12, 14) or the Montreal Cognitive Assessment (MOCA) (2), the role of cognitive disorders is not observed or is underestimated (1). Furthermore, cognitive disorders certainly do not all have the same impact and should be disentangled. Unilateral spatial neglect (USN) (4, 5, 7), cognitive resources (10), and executive function disorders (15–17) have been found to be associated with risk of falls.

Tools to measure balance are not designed to predict the risk of falls, and most do not include a cognitive assessment (18–22). The dual-task paradigm has been suggested to be associated with the Timed Up and Go test (23, 24). The scores built with a fall-predictive objective are multidimensional. To our knowledge, the Stroke Assessment of Fall Risk (SAFR) is the only assessment including a large section for cognition (3, 4).

The present study was conducted to help the whole medical, nursing, and rehabilitation team decide on the risk of free ambulation or not in a given patient after stroke, as soon as possible, before any neuropsychological assessment. We proposed to start from the identified daily comportment of the patient rather than a test or score. Among the main post-stroke cognitive disorders, we investigated those associated with high risk of falls because of increased risk-taking behaviour. This study had two parts: a survey of concerned professionals to identify the main cognitive disorders perceived as being risk-taking behaviour and a retrospective study investigating whether these cognitive deficits could distinguish fallers from non-fallers in patients during acute post-stroke rehabilitation.

## METHODS

### Survey of professionals

We used the Script Concordance Test, a tool validated more than 10 years ago, which aims to assess reasoning when interpreting clinical data in a context of uncertainty (25). To identify the main cognitive disorders perceived as being responsible for risk-taking behaviour by the professionals involved in a PRM or a neurological team, we surveyed physicians (PRM specialists, geriatricians, neurologists), therapists (physical, occupational and speech), and nurses by direct interview. The interview questionnaire proposed the following clinical case: “A 65-year-old patient with hemiplegia who walks with a cane after a stroke”. The main post-stroke cognitive disorders were pre-stated in a list of 9 symptoms easily identified by non-neuropsychologist professionals. Disorders were grouped according to their relation with gnosis (anosognosia and USN), language (motor and sensitive aphasia), executive function (precipitation, inattention, perseveration, apragmatism), and temporal orientation (confusion). The respondent was then asked about the impact of each symptom on the patient’s risk of falling on a 7-point Likert scale (greatly reduced, reduced, slightly reduced, unchanged, slightly increased, increased, greatly increased). A weighted score was used for responses that increased the risk of falling (slightly increased/increased/strongly increased) in order to assign a relative weight to the responses. Each “slightly increased risk of falling” response was weighted by 0.3, “increased risk of falling” response by 0.6, and “greatly increased risk of falling” response by 0.9. Then, for the 74 participants, a total of 66.6 expressed the higher perceived risk of each symptom.

### Retrospective study of patients

*Participants.* We retrospectively screened the files of all 120 patients who after a recent stroke were admitted to our PRM department from 1 January 2019 and were discharged before 30 April 2020. The study database was obtained from the electronic medical record (ORBIS Dedalus software, AGFA HealthCare, Mortsel, Belgium) and the fall episodes record (OSIRIS application, a system set up by our institution [AP-HP] for reporting and tracing any adverse event, including falls) of screened patients.

Inclusion criteria were inpatient rehabilitation after a recent stroke, whether the first or not, and acute care provided in a stroke, neurosurgical, or general intensive care unit. All patients had a balance disorder due to motor or sensory impairment, defined at admission by the inability to walk alone. Exclusion criteria were stroke in the context of another disease such as meningitis, COVID-19, known dementia, Parkinsonism, or other concomitant pathology that may affect balance.

### Data acquisition

Recorded data describing the patients were age, sex, stroke type (ischaemic/haemorrhagic), side and location of stroke, mean length of stay in the stroke unit and in the first PRM department, disease severity according to the initial National Institutes of Health Stroke Scale (NIHSS) in the stroke unit and the initial Functional Independence Measure (FIM) at admission in the PRM department, presence of anaesthesia or homonymous hemianopsia (HH), and any psychotropic treatment. Cognitive disorders scored as present or not were considered when they were provided with rehabilitation on the basis of the neuropsychologist’s or speech therapist’s report (anosognosia, USN, motor and sensitive aphasia, precipitation, inattention, perseveration, apragmatism, and confusion).

Falls were defined as an involuntary contact with the floor. Although a retrospective study, a real quantification of all falls occurring in the department was easy because each identified fall is systematically recorded by the team in the specific register of falls (OSIRIS). Hence the retrospective analysis is based on a prospective record. Nevertheless the causes of falls could not be analysed because they were not always recorded.

### Ethical approval

This study was approved by the “Comité d’Ethique de la Recherche, CER”, Institutional Review Board – IRB 00006477 – of HUPNVS, Université Paris Cité and AP-HP, 27 July 2023.

### Statistical analysis

Statistical analysis, performed with R 4.2.3 (R Foundation for Statistical Computing, Vienna, Austria), involved a retrospective study of data for patients. Continuous data are presented as mean (SD). Categorical data are presented as number (%). We compared fallers and non-fallers using the relevant measured data. Categorical variables were compared by χ^2^ and Fisher’s exact tests. Continuous variables were compared by Wilcoxon Mann–Whitney test. The threshold for statistical significance is set at *p* < 0.05. The effect size for each variable was calculated by using the appropriate formula: Cliff’s Delta for continuous variables and Cramer’s V for categorial variables (an effect size >0.5 being regarded as strong). We used multivariate logistic regression analysis to examine whether any of the significant associations between cognitive disorders and falling had any confounding factors. The latter associations were adjusted by possible confounders that were also found to be significantly associated with falls. The variables’ coefficients of the multivariate logistic regression were also used to calculate the adjusted odds ratios (ORs) and 95% confidence intervals (CIs) for the associations between cognitive disorders and falls.

## RESULTS

### Survey of professionals

All 74 contacted professionals responded: PRM specialists (10), neurologists (10), geriatricians (10), speech therapists (10), occupational therapists (12), physical therapists (10), nurses and caregivers (12).

Major cognitive disorders identified as associated with risk of falling were anosognosia (weighted score = 57.6/66.6), confusion (weighted score = 56.1), inattention (weighted score = 49.8), precipitation (weighted score = 49.5), and USN (weighted score = 49.5), contrary to perseveration (weighted score = 24.9) sensitive (weighted score = 13.5), or motor aphasia (weighted score = 9.3), and apragmatism (weighted score = 2.4).

There were few differences between professionals. The only difference was for the opinion regarding sensitive aphasia, which was considered to indicate risk of fall for 11/12 of nurses vs 6/30 physicians. Because the main topic of this study was not the detailed analysis of professionals’ opinions, no analysis was conducted.

### Retrospective study of patients

*Characteristics of patients (**Table I**):* After excluding post-stroke patients in the context of another disease (4 with SARS-CoV2, 4 other severe infectious diseases, 3 cardiac failure, 1 hypoglycaemia), we included records for 108 patients. In Table II stroke location may be seen. In total, 25 patients (23%) were fallers, and 11 of these fell multiple times.

**Table I:** Characteristics of patients and stroke (*n* = 108)

Age, mean (SD)	57.1 (10.8)
Sex, male/female, *n* (%)	72/36 (66.6 ; 33.3)
PRM department stay, days, mean (SD)	65.6 (63.7)
Lesion side, right/left, *n* (%)	51/57 (47.2 ; 52.8)
Stroke type, ischaemic/haemorrhagic, *n* (%)	77/31 (71.3 ; 28.7)
NIHSS, mean (SD) (*n* = 79)	9.55 (7.1)
FIM, mean (SD) (*n* = 106)	79.8 (30.3)
Clinical characteristics, *n* (%)	
Anaesthesia	54 (50)
Homonymous hemianopsia	24 (22.2)
Motor aphasia	34 (31.5)
Sensitive aphasia	14 (13)
Unilateral spatial neglect	32 (29.6)
Anosognosia	16 (14.8)
Inattention	54 (50)
Precipitation	23 (21.3)
Perseveration	42 (38.9)
Apragmatism	21 (19.4)
Confusion	7 (6.5)
Hypnotic treatment	2 (1.8)
Other psychotropic treatment	20 (18.5)

PRM physical and rehabilitation medicine; FIM: Functional Independence Measure; NIHSS: National Institutes of Health Stroke Scale.

**Table II:** Stroke location for non-fallers and fallers

Factor	Non-fallers *n* = 83	Fallers *n* = 25	Total *n* = 108
Ischaemic strokes			
Basilar artery	14	0	14
Posterior cerebral artery	1	2	3
Anterior cerebral artery	3	0	3
MCA S	17	5	22
MCA P	13	6	19
MCA T	12	2	14
ACh A	2	0	2
Haemorrhagic strokes			
Anterior	1	1	2
Superficial	10	4	14
Profound	8	5	13
Occipital	0	0	0
Brainstem	1	0	1
Cerebellum	1	0	1

MCA S: superficial middle cerebral artery; MCA P: profound middle cerebral artery; MCA T: total middle cerebral artery; AChA: anterior choroidal cerebral artery.

### Characteristics of fallers and non-fallers

*General characteristics:* Fallers and non-fallers did not differ in age, sex, type, side or location of stroke, or treatment (Table III).

**Table III:** Characteristics of non-fallers and fallers

Factor	Non-fallers	Fallers	Effect size
Patients, *n* (%)	83 (76.8)	25 (23.1)	
Age, mean (SD)	57.2 (13.9)	56.8 (12.5)	
Sex, male/female, *n*	56/27	16/9	
PRM department stay, days, mean (SD)*	48.5 (43.2)	122.4 (85.6)	–0.69
Lesion side, right/left, *n*	40/43	11/14	
Stroke type, ischaemic/haemorrhagic, *n*	62/21	15/10	
NIHSS, mean (SD) (*n* = 79)	8.8 (6.9)	12.7 (7.3)	–0.31
FIM, mean (SD) (*n* = 106)*	88.4 (27.1)	52 (22.6)	0.66
Anaesthesia, *n* (%)*	35 (42.2)	19 (76)	0.27
Homonymous hemianopsia, *n* (%)*	13 (15.7)	11 (44)	0.27
Cognitive characteristics, *n* (%)			
Motor aphasia	23 (27.7)	11 (44)	0.11
Sensitive aphasia	8 (9.6)	6 (24)	0.15
Unilateral spatial neglect*	17 (20.5)	15 (60)	0.35
Anosognosia*	3 (3.6)	13 (52)	0.57
Inattention*	31 (37.3)	23 (92)	0.45
Precipitation*	7 (8.4)	16 (64)	0.57
Perseveration*	23 (27.7)	19 (76)	0.41
Apragmatism*	12 (14.5)	9 (36)	0.20
Confusion*	3 (3.6)	4 (16)	0.19
Medical treatments, *n* (%)			
Hypnotic treatment	1 (1.2)	1 (4)	
Other psychotropic treatment	12 (14.5)	8 (32)	

* Significant at *p* < 0.05.

PRM: physical and rehabilitation medicine; NIHSS: National Institutes of Health Stroke Scale; FIM: Functional Independence Measure.

Falling was significantly associated with severity of stroke as measured by the FIM but not the NIHSS, but these latter data were available for only the 79 patients with ischaemic stroke. Falling was significantly associated with the presence of anaesthesia, HH, and length of rehabilitation stay. Hypnotic or psychotropic treatments were not associated with falling, but these data were not reliable because the exact treatment at the time of the fall could not be certified. On multivariate analysis, only FIM and length of stay were independently associated with the odds of falling.

*Cognitive disorders:* On univariate analysis, all cognitive disorders except motor or sensitive aphasia were significantly associated with the occurrence of falls, with a strong effect size for anosognosia and precipitation (Table III). After adjustment for length of rehabilitation stay, on multivariate analysis (Table IV), the odds of falling were associated with anosognosia, precipitation, inattention, perseveration, and USN. After adjustment also for FIM, anaesthesia, and homonymous hemianopsia, the odds of falling remained associated with anosognosia (OR 16), precipitation (OR 13.3), inattention OR (8.3), and perseveration (OR 4.9), but not USN.

**Table IV:** Association between cognitive disorders and falls

Cognitive disorders	Adjusted OR (CI)	*p*-value*
Anosognosia	**16 (2.4, 106.7)**	**0.004**
Precipitation	**13.3 (3.1, 56.2)**	**0.0004**
Inattention	**8.3 (1.6, 43.0)**	**0.01**
Perseveration	**4.9 (1.4, 18.0)**	**0.01**
Unilateral spatial neglect	–	0.08
Confusion	–	0.49
Apragmatism	–	0.48

* After adjustment for physical and rehabilitation medicine department stay, Functional Independence Measure, homonymous hemianopsia and anaesthesia. –: no adjusted odds ratio (OR) calculated because of a non-significant result. Bold *p*-values indicate significant at *p* < 0.05.

The whole regression model is provided in Appendix S1.

*Multi-fallers versus once fallers:* Among fallers, except for length of rehabilitation stay (149.8 [SD 70.78] vs 100.8 [SD 92.36] days), multi-fallers differed from once-fallers only in more frequent precipitation (*p* = 0.033). However, given the number of fallers (25), and thus the small sub-group of multi-fallers, the results of such an analysis could be questioned.

## DISCUSSION

This study focused on risk-taking behaviour as a major risk factor in falling after a recent stroke. The objective was to help neurological and PRM teams identify the role of the patient’s behaviour when a decision is taken concerning free ambulation or not. Cognitive functions were studied here as the main abilities allowing the danger, the risk of falling, in a given situation to be evaluated. We studied the possible relation between falls and certain clinical symptoms, easily identified by professionals taking care of patients, before any complete neuropsychological assessment. According to the opinion of neurological and PRM teams, major cognitive disorders identified as associated with risk of falling were anosognosia, confusion, inattention, precipitation, and USN. The professionals exhibited few differences among themselves, and a large consensus was evidenced. The retrospective study of patients revealed gnosis and executive disorders, specifically anosognosia and precipitation, but not USN, as independently associated with risk of falls after adjustment for length of rehabilitation stay and disease severity. Language disorders were confirmed to have no influence. Negative results regarding confusion should not be considered because the definition of this symptom is too vague to be reliable in such a study.

The key message of our study is that among post-stroke patients at risk of falling because of physical and balance deficiencies, the real risk of falls during their first hospital stay depends on their ability to identify the risks and adjust their comportment. In our department, as soon as patients are admitted, the medical, nursing, and rehabilitation team now assess their risk of falls according to patients’ physical abilities and their behaviour with the cognitive symptoms identified as indicating risk of fall. Patients are then allowed or not to walk alone, under human supervision, or with physical human help. This decision is checked at least weekly.

These results are congruent with the literature. Some cognitive disorders have been found to be associated with risk of falls but with contradictory results depending on how they were defined. Global assessment with tools such as the MMSE (8, 12, 14) or MOCA (2) may fail to find any relation of cognitive disorders with falls, as has been emphasized in healthy older adults (26). When cognitive disorders are disentangled, the specific role of some disorders seems clearer. Language disorders, even with impairment of understanding, were not associated with risk of falls (6, 8). USN, recognized as associated with poor balance (27), is also usually considered a risk factor in falls (4, 5, 7), but in the present study it was not an independent factor. The role of attentional and cognitive resources has been shown (12), as has the role of executive functions, with some test scores such as the treadmill test (15, 16), the Stroop test (15), or the specific “solving problem” item of the FIM (17). Such a role of executive functions but not other cognitive disorders has also been found in healthy older people for level of mobility (28) and risk of falls (26, 29). In older people with chronic cerebrovascular disease, poor balance has been found to be associated with executive disorders (30, 31) and the degree of physical activity (32), but falls were not monitored. Hence, impulsivity, problem solving, and attention may have a specific impact on balance and risk of falls and should be separated from other cognitive disorders.

Also, we did not observe any relation between the side of the lesion and the occurrence of falls. Many studies reported poorer balance after right vs left hemispheric stroke, but those interested in the real occurrence of falls did not find such a difference (6, 8, 10, 12). This finding points to the need to distinguish balance, usually studied in a static position or during a limited task, and the real occurrence of falls in daily life. Among a specific population of people with right hemispheric lesions, falls have been related to impaired judgement and lack of insight into their problem, and the usefulness of a measure of behavioural impulsivity has been emphasized (33). In our population, all fallers had a hemispheric stroke, which also highlights the crucial role of cognitive disorders.

The period of observation after stroke probably plays an important role. Most of the studies that failed to observe any relation between USN (8, 9, 14) or executive disorders (8,12) and falling reported populations at 6 or 12 months after stroke. This situation could explain why, in this phase, the relative risk of falls due to cognitive impairment seemed to be lower in a recent review (1.75) than that for impaired mobility (4.36) (34). The same kind of relative risk has been observed at the initial stage, with an OR of 4.6 for “impaired postural control while walking” and only 1.43 for “impaired cognition” but only assessed by the MOCA (1). As previously stated, the danger period for falls is the subacute stage, during the rehabilitation process, when recovery is not optimal but the solicitation of balance is high, as observed in our study and previously reported (17).

### Limitations

Despite the retrospective design of the study, it may have real value because of the prospective recording of all falls occurring in our department and the systematic neuropsychological examination of all patients. However, our study has some limitations because of its design. The first is that this is a single-centre study. The definition of the cognitive disorders could be questioned because they were not defined by any unique specific test but by the global conclusion of the neuropsychological examination and were considered relevant when the patient was provided with a specific rehabilitation programme. Then, because the cognitive disorders were defined by clinical symptoms easily identifiable by each member of the team, these data were easily collected. Another limitation is the measure of stroke severity. The initial NIHSS score was available for all ischaemic strokes, systematically collected in the stroke units, but was not available for haemorrhagic strokes treated in a neurosurgical or sometimes non-neurosurgical intensive care unit. The deficiencies and their consequences were assessed in the few days after PRM admission. All patients had balance disorder defined by the inability to walk alone. However, the measurement of motor deficiencies, which was not standardized, could not be assessed with a unique score. Anaesthesia, including hypoesthesia, was always mentioned as present or absent for the lower limb at least. Homonymous hemianopsia was defined as present or absent on the basis of clinical examination at the bedside. The evaluation of vertical perception would have been interesting, but for technical reasons it had not been evaluated for about half of the patients. Functional consequences of the deficiencies are usually assessed in the first days post-admission with the FIM, which was available in almost all files. By measuring the consequences of deficiencies, FIM gives a good insight into the severity of the deficiencies. The initial NIHSS and FIM showed the relative severity of the stroke, but in this population, without severe cases such as locked-in-syndrome, all patients at entry are expected to walk at least with help (with a human, orthosis, or device). The influence of hypnotic or psychotropic treatment could not be studied because we could not ensure the chronological concordance of the treatment and the falls. Length of rehabilitation stay could have influenced the frequency of falls because the longer the stay, the greater the risk, and also because patients with longer stay are usually those with more severe deficiencies, thus increasing the risk. Multivariate analysis showed the independence of the main cognitive disorders from length of stay.

The tools usually considered to predict the risk of falls have limitations. First, a distinction should be made between tools for balance and tools for risk of falls. The first tools are not designed to predict a risk of falls, and because most do not include cognitive assessment (18–22), they should not be used for this objective. Second, most of the tools developed to evaluate the risk of falls after stroke, or generally in older people, do not take into account the cognitive disorders influencing behaviour. Interesting cognitive items are still present in some multidimensional scores. The item “agitation” accounts for 1 in 5 points in the STRATIFY score (35), the item “mental status, overestimates/forgets limitations” accounts for 12% of the total Morse instrument (36), the item “altered awareness of immediate physical environment/impulsive/lack of understanding of one’s physical and cognitive limitation” accounts for 17% of the Johns Hopkins Hospital fall risk assessment tool (37) and the item “confusion/disorientation/impulsivity” accounts for 25% of the Hendrich II fall risk model (38). The relative interest of these scores for inpatient assessment is often questioned (39, 40). For stroke patients, Nyberg proposed a score with 11 items: one is devoted to visual neglect, representing 9% of the total (5). The best tool to our knowledge is the SAFR, specifically developed for inpatients in rehabilitation. It consists of 4 of 7 items for cognition (impulsivity, hemineglect, problem solving, and memory) accounting for 57% of the total (3). However, we consider that the cognitive disorders influencing behaviour are underestimated in these scores.

### Conclusion

Cognitive disorders do not all have the same impact on the risk of falls in patients after stroke. We confirmed the weight of disorders affecting behaviour previously described by some authors: gnosis, attention, and executive disorders. Assigning a level for the influence of cognitive disorders among a multi-dimensional score is challenging. In our opinion, the usual predictive tools largely underestimate risk-taking behaviour. We propose that the risk-taking behaviour of a patient, in the acute and sub-acute post-stroke phase, should not be considered as an additional risk factor for falls along with physical and general factors but rather as a multiplying factor applied to the others.

## Supplementary Material

RISK-TAKING BEHAVIOUR AND EXECUTIVE FUNCTIONS, A MAJOR COMPONENT OF THE RISK OF FALL FACTORS AFTER RECENT STROKE
