# Supplementary material for: RISK-TAKING BEHAVIOUR AND EXECUTIVE FUNCTIONS, A MAJOR COMPONENT OF THE RISK OF FALL FACTORS AFTER RECENT STROKE
Source: J Rehabil Med. 2024 Oct 22;56:40153. doi: 10.2340/jrm.v56.40153 (PMC11519672; doi:10.2340/jrm.v56.40153)
Supplement: RISK-TAKING BEHAVIOUR AND EXECUTIVE FUNCTIONS, A MAJOR COMPONENT OF THE RISK OF FALL FACTORS AFTER RECENT STROKE [file JRM-56-40153-s1.pdf]

Supplementary material has been published as submitted. It has not been copyedited, or typeset by Journal of Rehabilitation Medicine

Appendix SI

Logistic Regresssion Models

1. **Anosognosia**

Predictor	Estimate	SE	tStat	Adjusted OR	Lower_OR(95%)	Upper_OR(95%)	P_Value
Intercept	-1,227	1,18	-1,04	0,293	0,029	2,946	0,297
**Anosognosia**	**2,775**	**0,97**	**2,871**	**16,046**	**2,413**	**106,692**	**0,004**
PRM	4,127	2,5	1,65	62,011	0,461	8345,9	0,099
Anesthesia	0,088	0,75	0,117	1,092	0,25	4,768	0,907
FIM	-3,066	1,44	-2,12	0,047	0,003	0,79	0,034
Homonymous hemianopsia	0,647	0,72	0,899	1,909	0,466	7,821	0,369

2. Precipitation

Predictor	Estimate	SE	tStat	Adjusted OR	Lower_OR(95%)	Upper_OR(95%)	P_Value
Intercept	-0,747	1,15	-0,65	0,474	0,05	4,526	0,516
**Precipitation**	**2,587**	**0,74**	**3,516**	**13,296**	**3,143**	**56,252**	**0,0004**
PRM	3,699	2,35	1,576	40,399	0,406	4023,411	0,115
Anesthesia	0,305	0,75	0,406	1,356	0,311	5,911	0,685
FIM	-4,472	1,58	-2,83	0,011	0,001	0,253	0,005
Homonymous hemianopsia	-0,018	0,7	-0,03	0,982	0,249	3,88	0,98

3. Inattention

Predictor	Estimate	SE	tStat	Adjusted OR	Lower_OR(95%)	Upper_OR(95%)	P_Value
Intercept	-2,766	1,37	-2,02	0,063	0,004	0,923	0,044
**Inattention**	**2,116**	**0,84**	**2,516**	**8,295**	**1,596**	**43,094**	**0,012**
PRM	5,313	2,27	2,346	203,019	2,396	17201,894	0,019
Anesthesia	0,702	0,72	0,98	2,017	0,496	8,213	0,327
FIM	-2,714	1,43	-1,9	0,066	0,004	1,086	0,057
Homonymous hemianopsia	-0,115	0,68	-0,17	0,891	0,237	3,355	0,865

4. Perseveration

Predictor	Estimate	SE	tStat	Adjusted OR	Lower_OR(95%)	Upper_OR(95%)	P_Value
Intercept	-1,779	1,15	-1,55	0,169	0,018	1,592	0,12
**Perseveration**	**1,607**	**0,66**	**2,449**	**4,99**	**1,378**	**18,065**	**0,014**
PRM	5,664	2,1	2,698	288,2	4,708	17642,582	0,007
Anesthesia	0,632	0,71	0,894	1,881	0,471	7,509	0,371
FIM	-3,381	1,41	-2,4	0,034	0,002	0,537	0,016
Homonymous hemianopsia	-0,359	0,72	-0,5	0,699	0,172	2,845	0,617

5. Unilateral Spatial Neglect

Predictor	Estimate	SE	tStat	Adjusted OR	Lower_OR(95%)	Upper_OR(95%)	P_Value
Intercept	-1,194	1,12	-1,07	0,303	0,034	2,708	0,285
Unilateral Spatial Neglect	1,12	0,66	1,694	3,064	0,839	11,188	0,09
PRM	6,057	2,36	2,57	427,171	4,21	43347,333	0,01
Anesthesia	0,402	0,68	0,589	1,495	0,392	5,704	0,556
FIM	-3,627	1,39	-2,62	0,027	0,002	0,402	0,009
Homonymous hemianopsia	-0,331	0,7	-0,47	0,719	0,182	2,83	0,637

6. Confusion

Predictor	Estimate	SE	tStat	Adjusted OR	Lower_OR(95%)	Upper_OR(95%)	P_Value
Intercept	-0,879	1,07	-0,82	0,415	0,051	3,391	0,412
Confusion	0,735	1,22	0,602	2,086	0,19	22,867	0,547
PRM	5,845	2,14	2,736	345,622	5,247	22766,295	0,006
Anesthesia	0,468	0,68	0,69	1,597	0,422	6,037	0,49
FIM	-3,702	1,38	-2,67	0,025	0,002	0,372	0,007
Homonymous hemianopsia	0,023	0,66	0,035	1,023	0,282	3,717	0,972

7. Apragmatism

Predictor	Estimate	SE	tStat	Adjusted OR	Lower_OR(95%)	Upper_OR(95%)	P_Value
Intercept	-1,024	1,09	-0,94	0,359	0,043	3,033	0,347
Apragmatism	0,634	0,72	0,883	1,886	0,461	7,71	0,377
PRM	5,382	2,11	2,556	217,362	3,509	13462,821	0,011
Anesthesia	0,666	0,72	0,926	1,946	0,476	7,964	0,354
FIM	-3,689	1,38	-2,68	0,025	0,002	0,372	0,007
Homonymous hemianopsia	-0,011	0,66	-0,02	0,99	0,273	3,585	0,987
